# Can the rate and location of sessile serrated polyps be part of colorectal Cancer disparity in African Americans?

**DOI:** 10.1186/s12876-019-0996-y

**Published:** 2019-05-24

**Authors:** Mehdi Nouraie, Hassan Ashktorab, Nazli Atefi, Saman Azam, Taraneh Tarjoman, Edward Lee, Babak Shokrani, Ali Afsari, Akbar Soleimani, Adeyinka O. Laiyemo, Sanmeet Singh, Hassan Brim

**Affiliations:** 1 University of Pittsburg, Medical center, Pittsburg, PA USA; 2 Department of Medicine, College of Medicine, Washington, DC USA; 3 Pathology Department, Cancer Center, College of Medicine, Washington, DC USA; 4 Cancer Research Center and Department of Medicine, Howard University College of Medicine, 2041 Georgia Avenue, Washington, D.C, N.W. 20060 USA; 5 Division of Pulmonary, Allergy and Critical Care Medicine, Department of Medicine, University of Pittsburgh, Pittsburgh, USA

## Abstract

**Background:**

Up to 30% of colorectal cancers develop through the serrated pathway. African Americans (AAs) suffer a disproportionate burden of colorectal cancer. The aim of this study was to evaluate clinicopathological features of AA patients diagnosed with sessile serrated polyps (SSPs).

**Methods:**

We conducted a retrospective study of all colonoscopies (*n* = 12,085) performed at Howard University Hospital, from January 1st, 2010 to December 31st, 2015, of which 83% were in AA patients, (*n* = 10,027). Among AAs, pathology reports confirmed 4070 patients with polyps including 252 with SSPs. Demographic and clinical variables (i.e. sex, age, BMI, anatomic location, clinical symptoms, polyp size, and clinical indications were collected at colonoscopy.

**Results:**

In the AA population, the median age was 56 with interquartile range (IQR) of 51 to 62 years, 54% were female, and 48% had a BMI > 30. The most common reason for colonoscopy was screening (53%), whereas the prevalent reasons for diagnostic colonoscopies were changes in bowel habits (18%) and gastrointestinal bleeding (17%). The total number of SSPs among the 252 AA (diagnosed with SSPs) was 338. Of these, 9% (*n* = 29/338) had some degree of cytological dysplasia, primarily in the ascending colon (*n* = 6/42, 14%), Transverse colon (n = 2/16, 13%) and rectosigmoid (*n* = 19/233, 8%). About 24% of patients had more than 2 polyps. Most patients (76%) had distal SSPs (rectal and rectosigmoid), in comparison to 14% of proximal polyps and 10% of bilateral locations. Median SSA/P size for all locations was 0.6 cm.

**Conclusion:**

The prevalence of SSPs accounts for 6% of all polyps in AA patients and was diagnosed in 2.5% of all colonoscopies (*n* = 252/10,027), which is higher than Caucasians in the US. SSPs were predominantly located in the left side, as compared to published literature showing the predominance in the right side of the colon. Screening of CRC will have the chance to detect high risk SSA/P in this population.

## Background

Colorectal cancer (CRC) is the third most prevalent cancer in both sexes in the United States, and is the second leading cause of cancer-related deaths [1, 2]. A recent meta-analysis by Imperoale et al. found that there is no difference in the prevalence of advanced adenomas and advanced precancerous colorectal neoplasm between African Americans, Caucasians, and concluding that as such screening guidelines ought not to be changed based on patients’ race [3]. Among precancerous colon polyps, serrated polyps (previously grouped with hyperplastic polyps) have been classified as non-neoplastic lesions and were not indicated for endoscopic treatment. However, during the past thirty years, there have been reports suggesting neoplastic changes in some of these lesions [4–7]. Based on the World Health Organization (WHO), serrated polyps have been classified into three groups: Hyperplastic polyps (HPs), sessile serrated polyps (SSPs), and traditional serrated adenomas (TSAs) [8]. SSPs and TSAs are known to have malignant potential [9–11]. SSPs which still remain poorly defined, generally have a flat phenotype with a mucus cap, predominant right side location, and distinctive serrated saw-toothed morphology [12–14]. SSPs were reported to be more prevalent in women [14–17]. In contrast to the well-known adenoma-carcinoma sequence, the “serrated pathway” is not well studied. It has been recognized as a novel pathway accounting for up to 30% of colon cancers [5, 9, 18]. A proportion of SSPs may develop cytological dysplasia (SSA/P-D) which may rapidly progress to carcinoma [11]. However, differentiating SSPs from HPs by colonoscopy or histopathology remains difficult due to overlapping morphological and pathological features. Li et al., recently reported a positive role of education and training in finding SSA/PS cases by gastroenterologists (GIs). Hispanic and Asian backgrounds and smoking were linked to the type of adenoma and HP while SSA/P was not linked to race/ethnicity [19]. While colonoscopy is the best screening tool for CRC prevention, it has its own limitations due to its invasive nature which makes follow-up less desirable for surveillance purpose. Follow-up colonoscopy is necessary for 10–20% cases to prevent CRC within 6–60 months (interval colonoscopy) [20, 21]. Recent data has attributed SSA/Ps to both missed lesions and interval CRC development mostly in the right colon [11, 22, 23]. Additionally, the histopathological recognition of serrated polyps is still controversial for both experts and non-expert gastrointestinal pathologists [5, 20, 21, 23]. A recent study detected molecular markers specific to these type of lesions [24]. It identified previously undescribed, differential expression of miRNAs in SSA/Ps but not HPs, including miR135B, miR378A, miR548, miR9 and miR196B, suggesting that miRNAs, among other molecular markers, are good predictors of serrated neoplasia [24]. SSA/Ps are found in around 0.6% of patients undergoing routine screening colonoscopy, they account for 1–9% of all colonic polyps and 15–25% of all serrated lesions [9, 14, 25–27]. Histologically, they are characterized by architectural distortion, with basal crypt branching and dilatation (appear as flask or boot-shaped), serration, horizontal crypt orientation running to the muscularis mucosae, and mitoses in the upper levels of the crypts [8, 13, 28, 29]. African Americans bear a disproportionate share of the cancer burden,with the highest death rate and shortest survival of any racial or ethnic group for most cancers [2]. According to SEER 18 of National Cancer Institute, the number of deaths for AA from CRC was 52 per 100,000, compared to 40.6 for non-Hispanic Whites during 2009–2013 [30]. Despite these disparities, there are very few studies on clinicopathological and demographic features of serrated polyps in this high-risk population. The present study aims to assess SSA/Ps burden and associated features in an AA population.

## Methods

We conducted a retrospective study of all colonoscopies performed at Howard University Hospital, Washington D.C. from January 1st, 2010 to December 31st, 2015. The data collection was anonymous and was approved by Howard University Institutional Review Board (IRB-MED-79). The corresponding endoscopy and pathology reports were reviewed (*n* = 12,085). Of these, 83% (*n* = 10,027) were from African American patients. We reviewed all pathology reports containing “Colorectal Polyp” in their diagnosis resulting in 4070 cases. Using the same pathology database, we searched for colonoscopies reporting “Serrated Polyps” in their diagnosis and we extracted data for 252 patients with such diagnosis. Out of these, 10 patients had multiple colonoscopies. The included SSA/P cases were originally read and diagnosed by board-certified pathologists at Howard University Hospital. For the purpose of our study, these cases were reviewed again by two gastrointestinal pathologists blinded to initial diagnoses of the polyps. Clinicopathological data including histopathological diagnosis, clinical and demographical data, number of polyps, location, size, and type of each polyp from the reports were abstracted. Weight, height, and BMI were added using electronic medical record database. Location of the polyps were categorized as right-sided and left-sided. Right-sided lesions were defined as those proximal to the splenic flexure, whereas left-sided were defined as lesions within and distal to the splenic flexure [31]. Different symptoms or indications for colonoscopy were placed into categories: screening, abdominal pain, gastrointestinal bleeding, change in bowel habits, anemia, pelvic mass, positive fecal occult blood test (FOBT), and weight loss. Subjects with family history of colon cancer, Familial Adenomatous Polyposis (FAP), Lynch Syndrome, and history of Inflammatory Bowel Disease (IBD) were excluded.

### Pathological classification of serrated polyps and associated subtypes

Classification of polyps was extracted from pathology database (PowerPath) and reviewed by two expert GI pathologists. Polyp histology was categorized into six groups: Conventional adenomas (with or without high grade dysplasia), hyperplastic polyps, SSA/Ps (with or without high grade dysplasia), TSAs, mixed hyperplastic adenomatous polyp, and mixed adenomatous polyps with serrated architecture. SSA/Ps were diagnosed by architectural features based on four criteria: Crypt serration, crypt dilatation, crypts that run horizontal to the muscularis mucosae and crypts branching basally (appear mas flask or boot-shaped). Dysplasia was defined as either low or high grade. Advanced sessile serrated polyps were defined as polyps with cytological dysplasia and/or size ≥1 cm.

### Statistical analysis

Distribution of continuous variables was presented with mean (SD) or median (interquartile range). We used t-test to compare the continuous variables between different groups and chi-square (or fisher exact) test for comparing the distribution of categorical variables.

## Results

### Clinicopathological features and frequency of SSA/Ps

The clinical and histopathological characteristics of the sessile serrated polyps are summarized in Table 1. The total number of 252 African American patients diagnosed with SSA/Ps with or without dysplasia were included.

**Table 1:** Demographic, clinical and pathology features of SSA/P patients

AA Patients with SSPs	*N* = 252
Age mean, median (SD) (year)	57.6, 56.0 (9.0)
Female	136 (54%)
BMI	
< 25	35 (14%)
25–30	97 (38%)
> 30	120 (48%)
Reason for Colonoscopy	
Screening	134 (53%)
Diagnostic	118 (47%)
Number of All Polyps	
Specimen Number	604
Single	82 (33%)
Two	77 (30%)
> 2	93 (37%)
Number of SSA/P	
Specimen Number	338
Single	190 (75%)
Two	40 (16%)
> 2	22 (9%)
SSA/P Site	
Right	37 (15%)
Left	190 (75%)
Bilateral	24 (9.5%)
Unspecified	1 (0.5%)

The frequency of polyps of any kind in this cohort was 41% (4070/10,027), and the prevalence of SSA/Ps with or without dysplasia was 6% (252/4070). The median size for SSA/Ps regardless of location was 0.6 cm. Age-wise, 71% of the patients were between 50 to 65 years and 120 patients (48%) were obese with a BMI > 30. The most common reason for colonoscopy was screening (134, 53%). For patients with clinical symptoms (118, 47%), change in bowel habits (46/252, 18%) and gastrointestinal bleeding (43/252, 17%) were the more common symptoms.

The total number of all SSA/Ps in the 252 patients was 338 with an average number of 1.3 SSA/Ps per patient [190 patients (75%) had one polyp, 40 patients (16%) had two polyps, and 22 patients (9%) had more than two polyps]. More than half of the patients (150, 60%) had synchronous lesions with other types of histopathological features, with tubular adenoma being the most prevalent (175/604, 29%), followed by hyperplastic polyps (83/604, 14%), and mixed hyperplastic adenomatous polyps (7/604, 1%).

The total number of SSA/Ps with or without dysplasia were 338 polyps, in which 9% (*n* = 29/338, 29 SSA/Ps-D in 23 patients) had some grade of cytological dysplasia. Also 83 (33%) patients had advanced SSA/Ps (Table 2). Advanced SSA/Ps were defined as either large in size (> 1 cm) and/or multiple synchronous lesions and/or cytological dysplasia. There was no significant difference regarding age, BMI, and gender when compared to non-advanced SSA/Ps patients. Positive FOBT was significantly higher in patients with advanced SSA/Ps (*P* = 0.028).

**Table 2:** Clinicopathological features of the patients with Advanced versus Non-advanced SSA/Ps

	Non-advanced *N* = 169	Advanced *N* = 83	*P* value
Age, median (IQR)	56 (51–63)	57 (51–62)	0.9
Female	94 (56)	42 (51)	0.5
BMI, median (IQR)	29.3 (26.5–34.0)	29.8 (26.5–35.2)	0.7
Location, n (%)*			0.001
Left	134 (80)	56 (67)	
Right	26 (15)	11 (13)	
Bilateral	8 (5)	16 (19)	
Symptoms, n (%)			
Abdominal pain	19 (11)	12 (14)	0.5
GI bleeding	27 (16)	16 (19)	0.5
Screening	106 (63)	45 (54)	0.2
FOBT+	3 (2)	6 (7)	0.028
Heartburn**	15 (9)	9 (11)	0.6
Change in bowel habits	32 (19)	14 (17)	0.7
Bloating**	2 (1)	0	0.3
Anemia	7 (4)	1 (1)	0.2
Pelvic mass	1 (0.6)	0	0.5
Weight loss	6 (4)	7 (8)	0.1

* One patient had unknown location. **Reported as part of upper endoscopy

For location, 75% were in the left colon (Table 3). In our patient population, SSA/Ps were more likely to be left-sided (190, 75%, *P* = 0.06), with more frequency in rectum (107/338, 32%) and rectosigmoid (75/338, 22%). There was no significant difference between patients with right-sided polyps and left-sided polyps regarding age, gender, BMI, and clinical symptoms.

**Table 3:** Characteristics of patients based on location of SSA/Ps

	Left *N* = 190	Right *N* = 37	*P* value
Age, median (IQR)	56 (51–62)	56 (52–62)	0.4
Female, n (%)	102 (54)	23 (62)	0.3
BMI, median (IQR)	29.3 (26.5–34.8)	28.3 (25.8–31.6)	0.17
Symptoms, n (%)			
Abdominal pain	22 (12)	6 (16)	0.4
GI bleeding	27 (14)	10 (27)	0.053
Screening	119 (63)	20 (54)	0.3
FOBT+	7 (4)	1 (3)	0.8
Change in bowel habits	34 (18)	6 (16)	0.8
Anemia	7 (4)	1 (3)	0.8
Pelvic mass	1 (0.5)	0	0.7
Weight loss	8 (4)	3 (8)	0.3

The median age for patients with SSA/Ps was 56 (IQR = 51–62) which was similar to patients with HPs (56, *p* = 0.5; Table 4). On further comparison, the mean number of polyps detected for SSA/P patients was 1.3, in contrast to mean number of polyps detected in hyperplastic patients (1.2, *P* = 0.009).

**Table 4:** Characteristics of SSA/P and HP patients

	SSA/P N = 252	HP *N* = 142	P value
Age, median (IQR)	56 (51–62)	56 (52–62)	0.5
Female, n (%)	136 (54)	67 (47)	0.2
Location, n (%)*			0.06
Left	190 (76)	121 (85)	
Right	37 (15)	15 (11)	
bilateral	24 (10)	6 (4)	
Number of polyp, mean (SD)	1.3 (0.7)	1.2 (0.5)	0.009
Clinical symptoms, n (%)			
Abdominal pain	31 (12)	26 (18)	0.10
GI bleeding	43 (17)	24 (17)	> 0.9
Screening	151 (60)	85 (60)	> 0.9
FOBT+	9 (4)	5 (4)	> 0.9
Change in bowel habits	46 (18)	21 (15)	0.7
Anemia	8 (3)	6 (4)	0.6
Pelvic mass	1 (0.4)	0	0.5
Weight loss	13 (5)	10 (7)	0.4
Hemorrhoid	0	0	NA

*One patient had unknown location

## Discussion

The aim of this retrospective study was to evaluate clinicopathological features of African American patients diagnosed with SSA/Ps. Considering that these lesions are a new diagnosis without uniform classification criteria among pathologists and as such their prevalence is controversial, underestimated, and varies between 0 to 1.2% in the literature [14, 27, 32–35]. The prevalence of SSA/Ps in our study was 2.5% (252/10,027 colonoscopies) which is higher than that published in other groups. The reason for this difference might be due to our study population and also the inclusion of colonoscopies including diagnostic procedures rather than just screening tests as in other studies. Even when considering screening colonoscopies only (*n* = 134), the SSA/Ps rate in our population is at the upper edge of 1.33%, still higher than what is reported in other studies. Lower socioeconomic status is a risk factor for CRC as reported by DeNavas-Walt et al. African Americans in the U.S. have the lowest income [36]. Obesity rate in our patients was 48%. Obesity has been cited as a risk factor for serrated polyps mostly in the left side of the colon [37, 38]. It is worth noting that most SSA/Ps in our study were distal which give some credibility to the SSA/Ps-obesity link that are primarily in the left colon as in AA population. Other cited risk factors for serrated polyps are dietary habits and smoking which we did not collect for our study.

Colorectal cancer is highest among African Americans [1], and the prevalence of polyps in general is higher in African Americans who are usually diagnosed at advanced stages [39, 40]. However, there are no studies showing higher prevalence of sessile serrated polyps in this population. Wallace et al. conducted a research on the association of race, age and developing colorectal polyps in 2014, demonstrating that African Americans have a lower risk of developing serrated polyps, but they did not subcategorize the serrated polyps into SSA/Ps, HPs, and TSAs [37]. In another study, SSA/Ps prevalence was reported as lower in low-income AAs compared to whites (2 vs. 8%) [41]. In a prospective study of 190 Caucasian patients undergoing colonoscopy by a single operator, during a five month period at the Royal Brisbane and Women’s Hospital in Australia, Spring et al. reported that the prevalence of SSPs in their population was 13.8% of all colonoscopies and 9% of all detected polyps [42]. Kumbhari et al. in Australia, compared SSA/Ps prevalence between Caucasians and Chinese, and reported higher prevalence of SSA/Ps in Caucasians (7% vs. 2%) [43]. These two studies demonstrate a strikingly higher prevalence of SSA/Ps in the Australian Caucasian population, showing non-homogenous reports on SSA/Ps’ prevalence around the world. This emphasizes the need for more prospective research on this matter. In a more recent study in 759 serrated adenomas from Irish patients, McCarthy et al. reported that after a review of the cases 100 cases were reclassified, with the majority being changed from SSA/P to hyperplastic polyps, further stressing the need for meticulous review before stating final diagnosis, and while the majority of SSA/Ps were in the right colon (86.7%), most TSAs were in the left colon (85.7%) [44].

In our study, SSA/Ps account for 6% of all patients with detected polyps (*n* = 4070) which is in agreement with other studies demonstrating a rate of 1 to 9% [8, 13, 23, 26, 27]. Overall the average age in our population was 57.6 (± 8.9 years), and 12% of them were < 50 years old showing the advantage of starting screening colonoscopy at a younger age of 45, if not earlier as we previously recommended for African Americans [45, 46]. In contrast, the mean age at diagnosis for hyperplastic polyps was 62 (± 11 years) in previous reports [47]. SSA/Ps frequency was reported as very close in both sexes with a slight predominance in females, which is in agreement with our results (54% in females). The total number of SSA/Ps in our study was 338 in 252 patients, out of which 9% (*n* = 29/338) had some grade of cytological dysplasia. The average number of SSA/Ps per patient was 1.3, with 75% of patients having one SSA/P, 16% with two SSA/Ps, and 9% with more than two SSA/Ps with a range of one to four. In a study by Gurudu et al., synchronous polyps along with SSA/Ps were found to be present in 51% of the patients, with 49% being tubular adenoma, 10% tubulovillous adenoma and 40% hyperplastic polyps [48]. In our study, more than half of the patients (150, 60%) had synchronous lesions with other types of histopathological features, with tubular adenoma being the most prevalent (29%, 175/604), followed by hyperplastic polyps (14%, 83/604), and mixed hyperplastic adenomatous polyps (1%, 8/604). In general, the average number of all polyps per patient was 2.4 (604 polyps in 252 patients with a range of 1 to 9). The range of mean polyps per patient is various in different studies and is around 0.2 to 3.2 and is highly affected by operators and endoscopy performance [17, 42, 49]. Since SSA/Ps are a new diagnosis, there is no credible data on the mean number of SSA/Ps per patient and it appears to be significantly underdiagnosed in clinical practice. Its diagnosis is highly variable between practitioner endoscopists and pathologists [11, 32, 33, 50–52]. The flat appearance and mucinous feature of these polyps make them hard to detect at endoscopy and their characteristic histologic features and location in the crypt base rather than the mucosal surface make them difficult to dissociate from other serrated lesions [5, 27, 53, 54]. Lack of uniform pathologic criteria amongst pathologists may account for the misdiagnosis of serrated polyps [5, 20, 21, 23]. The other important reason for underdiagnosis is bowel preparation before endoscopy and the quality of endoscopy [11, 32, 33, 50–52]. It has been suggested that the inability to detect and resect SSA/Ps might be one cause of the lower detection of these lesions leading to the development of interval cancers [20, 54]. There is a hypothesis that patients with SSA/Ps, have a higher chance of developing synchronous serrated polyps, metachronous adenoma and CRC [32, 38, 55]. But it is unclear, whether this is due to genetic alterations in these patients making them more susceptible to develop neoplastic polyps or due to some genetic/epigenetic alterations specific to these lesions. Burnett-Hartman et al. reported that age, obesity, NSAID use, and family history of colorectal cancer have stronger association with synchronous adenoma and serrated polyps than those with only 1 type of polyp [38]. Since these are known risk factors for colorectal cancer, more vigilance to patients with SSA/Ps is recommended.

Some SSA/Ps may develop different grades of cytological dysplasia, which have greater chances of becoming cancerous. In recent studies, the range of SSA/Ps with dysplasia was reported to be around 0.3 to 25% [14, 27, 32, 56, 57]. The rate of dysplasia in our AA population was 8.57% when considering total SSAPs (29/338) and 9.1% when considering patients with dysplasia (23/252). Average size for SSA/Ps is typically more than 5 mm [28, 34, 56–59]. There are some suggestions on larger size SSA/Ps with increasing age or developing dysplasia [56], but in our data there is no difference in the mean size of SSA/Ps in all locations and in all age ranges (6 mm).

The most common reason for colonoscopy in our study was screening (134, 53%). For patients with clinical symptoms (118, 47%) undergoing diagnostic colonoscopy, changes in bowel habits (46/252, 18%) and gastrointestinal bleeding (43/252, 17%) were the most common symptoms. Some studies have shown that asymptomatic AAs have a greater risk of developing polyps and CRC compared to whites [40, 46, 60]. In recent studies, clinical symptoms associating with SSA/Ps were: anemia, blood per rectum, weight loss, and abdominal discomfort [26, 34, 35]. However, the most common reason for colonoscopy in these patients was screening in most studies which is in agreement with our results [11, 35, 46, 59]. Clinical symptoms associated with colorectal adenomas are blood per rectum and anemia due to polyp bleeding, and symptoms like diarrhea, constipation, and abdominal pain due to polyps’ direct compression to structures around it. But sessile serrated polyps are flat in shape, hence they may not present with such clinical symptoms. In our patient population, SSA/Ps were more likely to be distal (190, 75%), with more prevalence in the rectum (107/338, 32%) and rectosigmoid (75/338, 22%). Patients with right-sided polyps were significantly older (mean age of 60 years in comparison to 56 years, *P* < 0.05). As such, the prevalence of left-sided SSA/Ps in younger patients might point to a new etiology of CRC in AAs and might be a participating factor in the overall CRC disparity in this population.

The limitation of this study was that it was a retrospective study; hence, we could not collect information on bowel preparation quality, diet and smoking habits that are factors in the etiology and detection of such flat lesions.

Due to either small sample size studies [17, 61], or non-categorization of SSA/Ps with the current definition [26], SSA/Ps remain underdiagnosed. The flat appearance of these polyps or endoscopist experience might not be the reason for this discrepancy, since they were predominantly detected in the left side of the colon in previous studies. There is a hypothesis that suggests that some of the SSA/Ps arise from hyperplastic polyps [5, 62, 63]. There might be some interobserver error misdiagnosing some of the SSA/Ps as HPs, or vice-versa. However, a better understanding of the molecular features of serrated lesions will facilitate a more objective approach for discriminating these lesions, even though they seem to share many features. [10, 11, 23, 64, 65]. Although there are some recent findings [18, 23] on gene alteration to differentiate these two types of polyps [9, 11, 12, 28, 62, 66], further understanding of the serrated lesions at the genetic level is needed in addition to a prospective study in the African American population.

## Conclusion

The prevalence of SSPs accounts for 6% of all polyps in AA patients and was diagnosed in 2.5% of all colonoscopies (*n* = 252/10,027), which is higher than Caucasians in the US. SSPs were predominantly located in the left side, as compared to published literature showing the predominance in the right side of the colon. Screening of CRC will have the chance to detect high risk SSA/P in this population.
